# Controlling Phase in Colloidal Synthesis

**DOI:** 10.1021/acsnanoscienceau.3c00057

**Published:** 2024-02-29

**Authors:** Emma J. Endres, Jeremy R. Bairan Espano, Alexandra Koziel, Antony R. Peng, Andrey A. Shults, Janet E. Macdonald

**Affiliations:** Department of Chemistry, Vanderbilt University, 2301 Vanderbilt Place, Nashville, Tennessee 37235, United States

**Keywords:** phase, polytype, polymorph, chalcogenide, pnictide, transition metal, synthesis

## Abstract

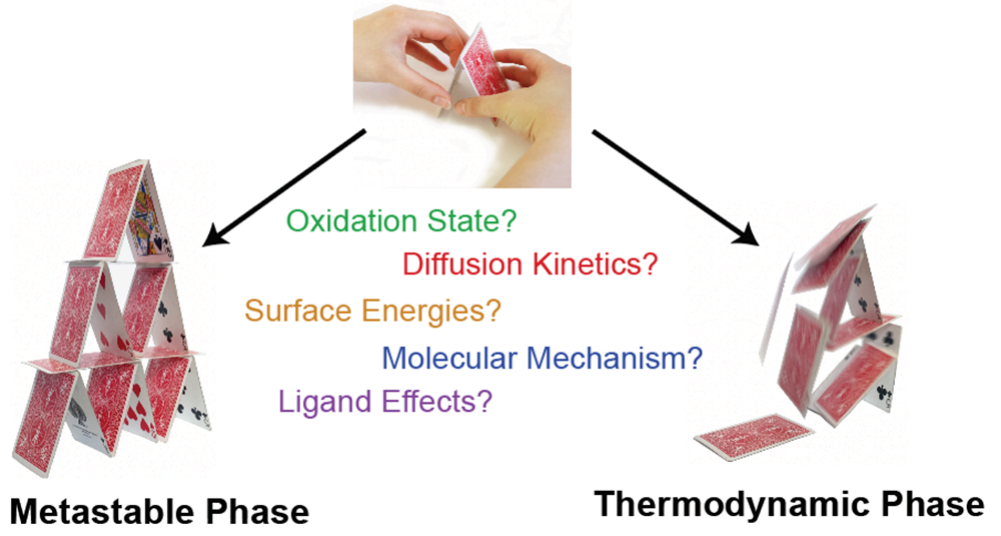

A fundamental precept of chemistry is that properties are manifestations of the elements present and their arrangement in space. Controlling the arrangement of atoms in nanocrystals is not well understood in nanocrystal synthesis, especially in the transition metal chalcogenides and pnictides, which have rich phase spaces. This Perspective will cover some of the recent advances and current challenges. The perspective includes introductions to challenges particular to chalcogenide and pnictide chemistry, the often-convoluted roles of bond dissociation energies and mechanisms by which precursors break down, using very organized methods to map the synthetic phase space, a discussion of polytype control, and challenges in characterization, especially for solving novel structures on the nanoscale and time-resolved studies.

## 1 Introduction

The binary phase diagrams demonstrate a diverse array of late metal chalcogenides and pnictides of varying composition and crystal structures. For instance, there are (at least) nine known crystalline phases of iron sulfides, ten copper sulfides, and ten nickel phosphides. The selenides and arsenides are less numerous, but several compositions and polymorphs are known for each metal. While a few of the known phases are purely synthetic, the diversity and complexity of the phase diagrams that nature has achieved is inspiring. These compounds have a myriad of potential technological applications because of their diverse electronic (semiconductors, semimetals, metals), optical (band transitions and plasmonic), magnetic, and catalytic properties. Synthetic chemists have not accessed all these nanocrystal phases, and without a fundamental understanding of phase control, more synthetically challenging targets such as hybrid, doped, or alloyed nanostructures cannot be readily pursued. In this Perspective, we examine the validity of long-standing concepts and fresh ideas in the pursuit of phase control in colloidal nanocrystal synthesis.

Nature’s most prolific solid state inorganic chemist, geology, has the advantages of a wide variety of temperatures up to thousands of degrees, variable pressures, variable metal to chalcogenide or pnictide ratios, and cooling rates covering seconds to thousands of years to achieve the vast diversity of stoichiometric phases and metastable polytypes. These extreme conditions, when compared to the typical length of a Ph.D. program, exclude the pursuit of a universal geo-mimetic^1^ tactic in bottom-up syntheses of nanocrystals.

At our disposal are powerful chemical tools that geology does not have: highly controllable and diverse organochalcogenide and organopnictide chemistry that can tune both the inherent reactivity of the precursors and the decomposition mechanisms on metals to yield free chalcogenide or pnictide. In some cases, this may make chemists more powerful than geology; a few metal chalcogenide phases have only been synthesized in nanocrystalline samples from bottom-up and nanocrystal cation exchange processes.^2−5^ This means that not only is there the possibility to achieve the diverse phases of the geologic record, but there is room to further expand the known phase diagrams with materials that have yet completely undiscovered properties.

Our colleagues in synthetic organic chemistry have long been inspired to accomplish the chemical diversity of natural products.^6−8^ A well-trained organic chemist can see the structure of a new natural product and readily come up with a reasonable retro-synthetic pathway to achieve that goal. In contrast, as inorganic nanocrystal chemists, we do not have the synthetic toolkit to imagine a synthesis that selects for one phase over another, nor rationally tweak a “failed” reaction to achieve a goal.

**Scheme 1:**
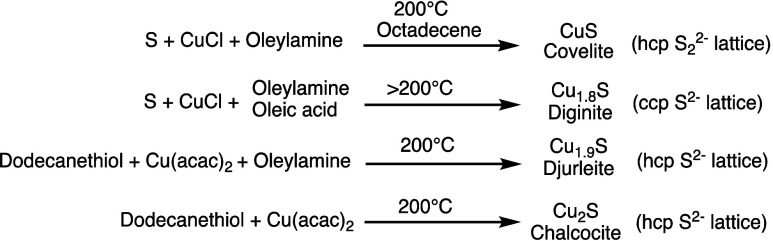
Selected Reported Colloidal Syntheses to Several Copper Sulfide Phases From refs (9−12).

For example, Scheme 1 captures several literature preparations for different copper sulfide phases using either dodecanethiol (DDT) or oleylamine-sulfur mixture. The observations are phenomenological and without trends that can be applied to other metals.

How can serendipitous discovery, stochastic iterations, and phenomenological observations be transcended to a rational pattern that can be exploited for nuanced phase control? How can the same diversity in crystalline phase be achieved in bottom-up syntheses as in the geological record?

We can be inspired by the long history of organic total synthesis and the careful study and exploitation of both kinetic and mechanistic considerations of solution chemistry that control products. In this Perspective, we will discuss the work being performed on metal chalcogenides and pnictides that specifically target phase control. Both prevailing and new approaches to stoichiometric and polytype phase control will be discussed.

## 2 Synthetic Challenges in the Metal Chalcogenides

In metal sulfides and selenides, the formal oxidation state of both the metal and the chalcogen are variable. With the exception of the coinage metals (Cu^1+^, Ag^1+^, Au^1+^), almost all the late metal chalcogenides find the metal in the (II) oxidation state. The spinel structures, such as Fe_3_S_4_, Ni_3_S_4_, and others, contemporaneously host both M^2+^ and M^3+^ ions.

In other structures, it is the chalcogenide that is oxidized in the form of persulfide S_2_^2–^. Pyritic (*cubic-*FeS_2_) and marcasitic (*orthorhombic*-FeS_2_) structures feature metal ions in octahedral holes with S_2_^2–^ in fcc and hcp packing, respectively. Similar structures are seen in vaesite (NiS_2_), cattierite (CoS_2_), as well as other metal sulfides, selenides, and tellurides. Multiple oxidation states of chalcogenide can be supported in the same structure. Covellite (CuS), spionkopite (Cu_1.4_S), and yarrowite (Cu_1.2_S) have close packed persulfide (S_2_^2–^) layers interspersed at differing intervals with S^2–^ layers.

Some of the more common sulfur reagents include thiourea,^13,14^ elemental sulfur,^15^ sodium sulfide,^16^ thioacetamides,^17^ carbon disulfide,^18^ oleylamine-sulfur(thioamides),^19^ dithiocarbamates,^20,21^ thiobiurets,^22^ thiols,^23^ and thioethers.^8^ Common selenium precursors include elemental selenium in octadecene or amines, selenourea,^24^ aromatic and dialkyl diselenides.^25^ Tellurium reagents include didodecylditelluride,^26^ elemental tellurium,^27^ and TeO_2_ in reducing environments.^28^ Being so low on the periodic table, tellurium’s soft nature and highly negative reduction potential makes it unlikely to react with a metal at high temperatures common to nanocrystalline syntheses.^26^ In these conditions, tellurium precursors will often decompose into Te(0) particles.

Metal oxides are generally formed through reactions with atmospheric oxygen or water. Studies on the phase control of VO_2_ by the Knowles group have shown exquisite sensitivity to the pH and concentration of water.^29,30^ In some cases, acetylacetonato ligands have been identified as an oxygen source in colloidal synthesis.^31,32^ Controlling phases in oxides is a large field with different approaches than those of the lower chalcogenides. Typically, high pressures are needed to crystallize the products, and pressure becomes an important experimental factor in phase control. Autoclave or “bomb” reactors prevent aliquot studies making following growth and nucleation particularly challenging. Phase control in the oxides is a topic deserving of its own perspective and will not be discussed further here.

Other than oxygen, the chalcogenides are highly flexible in oxidation state in solution going from −2 up to +6. Even under reduced conditions, it is important to remember that when elemental chalcogenides are present, oxidation state can be fractional between 0 and −2; polysulfides, -selenides, and -tellurides have the structure (X_*n*_^2–^), gaining and losing length to their chains and rings to modify the formal shared oxidation state of the oligomer. A common reviewer’s refrain is to question the role of oxidation state of the metal precursor; however, when there is excess chalcogenide in solution, it is questionable if the starting metal oxidation state is important, because of the flexibility in the chalcogenide oxidation state. This is mere speculation on our part, and this is a place for further investigation. There is some evidence to the contrary; for example, Plass et al. showed that changing reducing power of dodecanethiol by adding oleic acid, changed the phase of copper sulfide from chalcocite to tetragonal chalcocite (Cu_2_S), digenite (Cu_1.8_S), and covellite (CuS).^33^

One of the greatest challenges is simply understanding how the different phases are related to one another structurally. In some cases, the structures can simply be described by close packed anion structures with cation filling. In others, distortions can greatly decrease the symmetry, increasing the unit cell to an unwieldy size. For example, pyrrhotite 5C (Fe_9_S_10_) has a unit cell with 198 atoms.^34^ Any attempt to intuitively discover relationships between phases makes one want to close VESTA^35^ and throw one’s hands up in frustration.

But all is not lost. In many cases, the nuanced perfection demanded by crystallographers that occludes broader structural features and relationships can be simplified. Differences in the pattern of cation filling or vacancies can create a set of related crystal structures with what can appear at first glance as very different unit cells; yet the structures are similar if one were to “squint”. For example, the 198 atom unit-cell pyrrhotite 5C belongs to a larger family of (Fe_1–*x*_S) pyrrhotite structures which includes troilite as the stoichiometric end member (FeS). Despite the complexity, all structures have approximately the NiAs structure (hexagonally close packed (hcp) in the anion with the cations in Octahedral (O_h_) holes) but with differing vacancy patterns.

**Figure 1:**
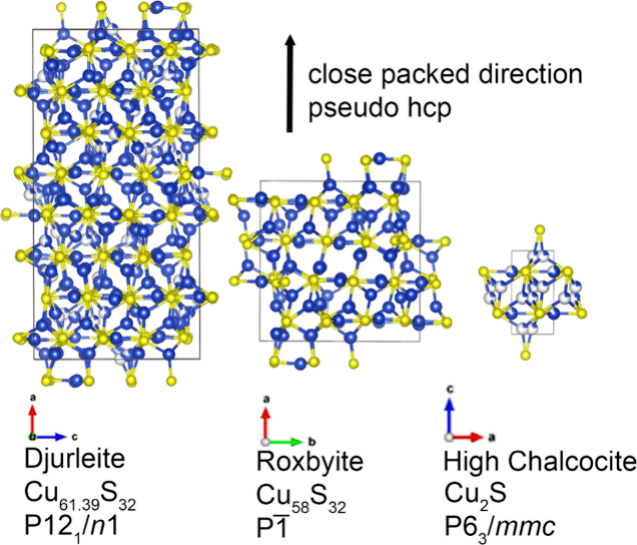
VESTA^35^ renditions of djurleite,^36^ roxbyite,^36^ and high chalcocite.^37^ Despite the sometimes large unit cells and low symmetry, all three can be described as pseudo-hcp in sulfur (yellow) with differing hole fillings and vacancy patterns of copper (blue). Low chalcocite (not shown) is similarly complex to djurleite and roxbyite, but above ∼103.5 °C, the coppers become mobile, and all three structures simplify to high chalcocite.^36^

Similar examples of structural complexity come from the phases of the Cu_2–*x*_S family. Digenite, anilite, and geerite (Cu_1.6–1.8_S) are all approximately cubic close packed (ccp) packed in sulfur while djurleite, roxbyite, and chalcocite (Cu_1.78–2.00_S) can all be described as approximately hcp packed in sulfur (Figure 1). They differ in the exact pattern of cation hole filling and number and position of copper vacancies with some mild distortions from a perfectly packed lattice, which leads to enormous unit cells in their full crystallographic solutions. In the hcp lattice structures, at the synthetic temperature of most nanocrystal syntheses, the crystallographic copper positions become moot, because the copper ions become mobile above about 103.5 °C in bulk and at even lower temperatures in nanocrystals.^38^ Our group often finds it instructive, therefore, when presented with a large intimidating unit cell, to seek older literature often from the 1930s to 1970s and other sources that describe crystal structures in more approximate manners, when trying to understand broad-strokes relationships between phases. One must be careful though that the structures were not truly misassigned. In discussion among ourselves and in manuscripts, we usually describe structures simply by their pseudo-close-packing (ccp or hcp), type of hole filling (O_h_, T_d_, or trigonal), and hole filling ratio (all filled, partly filled). Lately, we even often avoid common mineralogical descriptors such as “rock salt” or “wurtzite” in group meetings to make sure even new group members can follow the discussion. When writing papers, we will still use the mineralogic descriptor at least in passing, acknowledge any intentional simplifications, and provide references for the full structures.

There are, of course, many structures that defy simplification into close packed anions with cation hole filling. A great example is millerite (NiS), which has five-coordinate, square pyramidal coordination of the Ni throughout. While we synthetic chemists can tackle some of the simpler crystal families intuitively, we must recognize our limitations. Very exciting is the recent computational research of the Van der Ven group, who are finding a way to quantify and map similarities between phases and transformations between them.^39−41^ Their work to date has mostly focused on intermetallics and alloys; however, their approaches may be invaluable to polar compound materials. This is potentially a rich avenue for phase control and understanding how and why some phases convert into others, especially when complex structures are involved.

## 3 Confounding Bond Dissociation Energies (BDEs) and Mechanism

The most utilized approach for rationally controlling phase in the metal chalcogenides is correlating the bond strength of organochalcogenide precursors with the resultant phase. The presumption is that precursors that have weak chalcogen-carbon bonds will release more chalcogenide quickly (if not a limiting reagent), yielding chalcogen rich phases, influencing the production of kinetic vs thermodynamic polytypes (more later).

**Figure 2:**
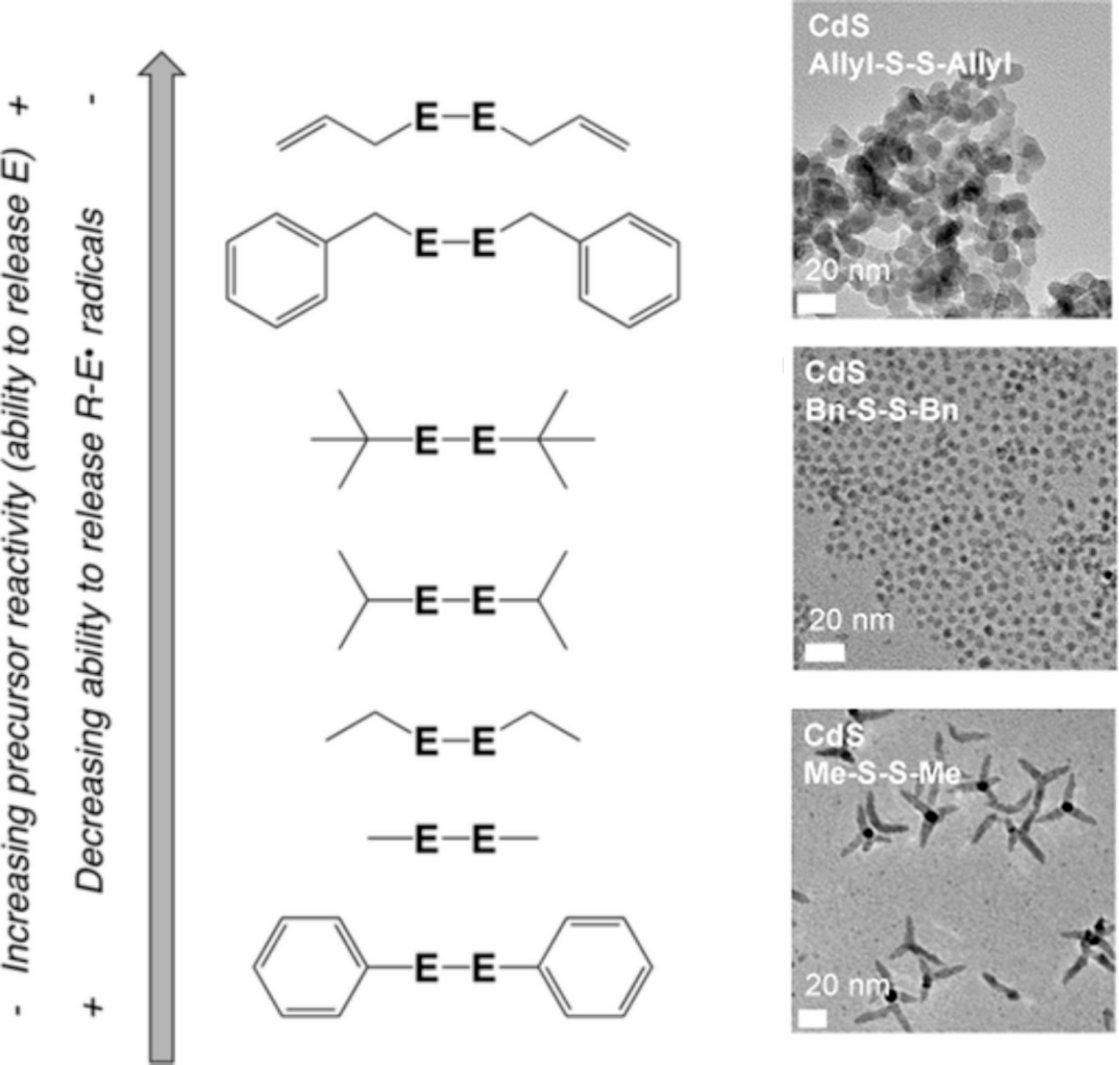
Dichalcogenides that more easily release E (Se or S) gave spherical hexagonal CdS or CdSe with the wurtzite crystal structure, while those that release the chalcogenide more slowly yielded tetrapods. Tetrapods have zinc-blende cores with hexagonal arms, suggesting that phase is dependent on the bond dissociation energy of the chalcogenide precursor. Adapted from ref (42). Copyright 2013 American Chemical Society.

As an example, the Vela group employed a series of organo-dichalcogenides, including dialkyl-, diphenyl-, and dibenzyl-disulfides and diselenides, as precursors and studied the size and shape control of CdSe and CdS (Figure 2). In their experiments, they found the determining factor in precursor reactivity is the carbon-chalcogenide bond dissociation energy (BDE), while the chalcogen-chalcogen BDE remained constant. In most cases, the thermodynamic wurtzite CdSe and CdS resulted, yet with precursors with the strongest C–S or C–Se bonds, tetrapod shapes resulted. Tetrapods contain zinc-blende cores with wurtzite arms, suggesting phase control is determined by the BDEs of the organochalcogenide precursor.^42^ (Figure 2)

**Figure 3:**
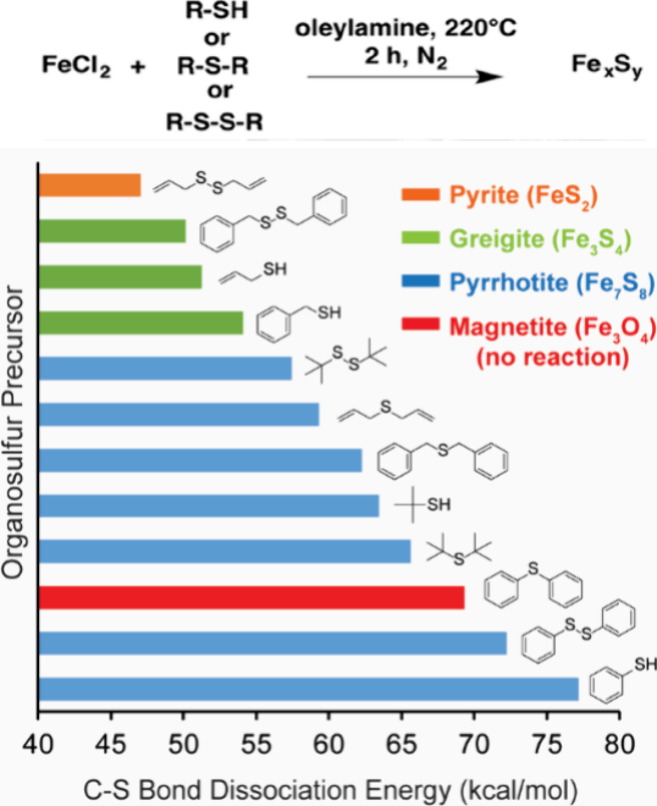
Sulfur content of the phase of Fe_*x*_S_*y*_ is correlated with the C−S bond strength of the organosulfur precursor. Adapted from ref (8). Copyright 2017 American Chemical Society.

Inspired by the observations of the Vela group, our group showed a correlation between C–S bond strength, calculated from density functional theory (DFT), of the organosulfides and the sulfur content of the resultant phase in iron sulfides; the weakest bonds gave the most sulfur-rich phases.^8^ These trends in bond dissociation energy are promising, but is it really that simple? Do more reactive chalcogenide precursors lead to the more chalcogenide-rich phases? Or are there far more complex mechanisms at play that have been undervalued? More carefully examining the molecular chemistry precluding nanocrystal formation, we determined that one of the sulfur sources, diallyl disulfide, has unique decomposition chemistry that plays a leading role in the phase control.^8^ While there can be a correlation between bond strength and the stoichiometry of the metal chalcogenide phase that results, differing decomposition mechanisms can derail these simplistic interpretations (Figure 3).

Synthesis of ternary and quaternary chalcogenides requires special mention because their mechanism of formation that controls phase has been partially identified in some cases. Organo-dichalcogenides have a propensity to give unique metastable hexagonal phases. The Brutchey group employed dimethyl-, dibenzyl-, and diphenyl-diselenide precursors and showed that the selenium precursors with weaker C–Se bond strengths such as dibenzyl and dimethyl diselenide resulted in the thermodynamic chalcopyrite CuInSe_2_, while stronger C–Se bonds in the diphenyl diselenide resulted in a novel metastable hexagonal CuInSe_2_ phase.^43,44^ A similar wurtzite-like polymorph of Cu_2_ZnSnSe_4_ was found through similar routes.^45^ The Ryan group found that selenium powder in oleylamine gave cubic Cu_2_SnSe_3_, whereas using diphenyl diselendie (Ph_2_Se_2_) gave the hexagonal phase. Through further system control, phase pure crystals, or heterostructures of these two phases could be produced.^46^

In many cases, it has been discovered that changing the chalcogenide reagent, changes the binary phase that forms first, which templates the crystal structure for cation exchange and growth of the ternary or quaternary structure. Further examination of the intermediates in the synthesis of wurtzite-like CuInSe_2_ showed that Ph_2_Se_2_ as a selenium source nucleates umangite Cu_3_Se_2_ which then transforms into wurtzite-like CuInSe_2_ product in a secondary step. Umangite has an approximate hcp stacking of the Se-anions, and this established structure templates the hcp Se lattice in the wurtzite-like phases. Adding to this interpretation, Leach et al. monitored the formation of a similar metastable wurtzite-like CuInS_2_ and found that the important step to producing this hexagonal phase was the formation of a hexagonal Cu_2_S intermediate upon which ion exchange occurs.^47^ If instead an indium sulfide forms first, the result is the more stable chalcopyrite phase.^48^ While synthesis of ternary copper chalcogenides undergo this two-step mechanism, what is not known is how broadly this applies to other ternary materials. It is likely many do not, but still, by studying early stages of crystal growth, it may be possible to find new routes to metastable ternaries beyond the copper family. Phase control in ternary metal chalcogenides have an extra level of mechanistic considerations of a potential two stage processes. (Deeper discussion of cation exchange, which is another approach to metastable phases of nanocrystals is outside the scope of this perspective.)

Bond dissociation energy is most often evoked when considering the chalcogenide reagent; however, similar arguments can be made for the metal source. Penk et al. utilized didodecyl ditelluride as a precursor to produce late transition metal tellurides including FeTe_2_, CoTe_2_, NiTe_2_, RuTe_2_, PdTe, PdTe_2_, and PtTe_2_. In this case, the identity of the counterion to the metal, being chloride, bromide, iodide, acetate, or triflate, was instrumental in successful production of the product metal tellurides. Normally, triflate should behave similarly to bromide or iodide, but in this instance, triflate salts were far less reactive than expected. While halides can do one- or two-electron chemistry, trifoliate can only do two-electron chemistry; thus, it was inferred that one-electron chemistry is underpinning the formation of metal tellurides from didodecylditelluride. The reactivity of all the metal centers correlated with the radical stability of the counter-species, with metal iodides being the most reactive.^26^ More recently, the group of Hernández-Pagán similarly found the phase control of the MnS and MnSe systems was sensitive to the halide (F^–^, Cl^–^, Br^–^, I^–^) counterion of the manganese precursor employed during synthesis.^49^

**Scheme 2:**
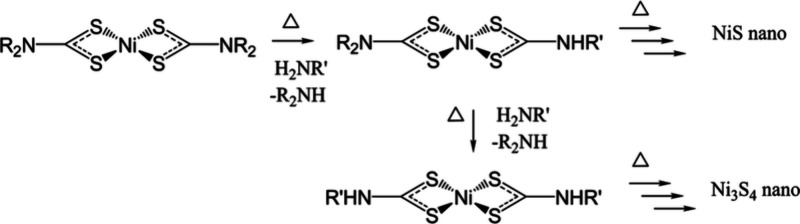
Nickel Thiocarbamate Ligands Can Decompose in Two Different Mechanisms Depending on the Concentration of Primary Amine in Solution; NiS or Ni_3_S_4_ Nanoparticles Result Reprinted with permission under a Creative Commons 3.0 Deed License from ref (20). Copyright 2016 Royal Society of Chemistry.

Some studies do not take BDE into account and instead approach phase control from a purely mechanistic viewpoint. For example, the Hogarth group was able to identify two competing routes of decomposition in nickel(II) dithiocarbamate precursors leading to differing phases of nickel sulfides. Without added primary amines, the reaction did not progress. Primary amines in solution caused exchange of the NBu^i^_2_ on the dithiocarbamate precursor to NHR. One exchange led to a NiS product. Excess amine caused two exchanges and gave a mixture of NiS and Ni_3_S_4_. (Scheme 2).^20,21^ Further manipulation of the system with temperature, amine concentrations and added thiuramdisulfide (a neutral oxidized dimer of dithiocarbamate) allowed for the production of α-NiS, β-Nis, Ni_3_S_4_, or NiS_2_.^20^

As further evidence of the role of mechanism in phase control, we diligently studied the formation of copper selenides using diphenyl- and dibenzyl-diselenide, which have differing C–Se and Se–Se BDEs. ^77^Se NMR provided a handle to study the molecular transformation in solution. We found that there are two underlying decomposition mechanisms of dibenzyl diselenide and diphenyl diselenide promoted by copper ions—Cham-Lam-like and hydrogen peroxide-like—that were dependent on the selenium precursor and the presence of oleylamine solvent. Decomposition mechanism was the most important determinant of phase in this system where all eight natural phases of copper selenide were synthesized.^25^

The extreme solution temperatures of colloidal synthesis and the presence of metal ions are magic pixie dust to organic chemistry, and all sorts of unexpected transformations can occur either as side reactions or in the course of a desired reaction.^20^ For example, it has been shown that commonly used ligands and solvents once thought of as “benign,” such as octadecene, steryl- and oleylamine, steric and oleic acid, and trioctylphosphine react extensively *in situ* with alkyl selenols at 155 and 220 °C. The results are selenoethers, diselenides, H_2_Se, selenoesters and trialkyl phosphine-selenides. The only truly “benign” solvent tested was dioctyl ether. Changing the nature of the selenium reagent **in situ** consequently changed the nanocrystalline product phase in the synthesis of Cu_2-x_Se.^50^ Another example is seen in the transformation of FeS to FeS_2_. Octadecylamine ligand reacts with elemental sulfur to produce H_2_S gas, which, in turn, is responsible for the solid transformation.^51^

**Scheme 3:**
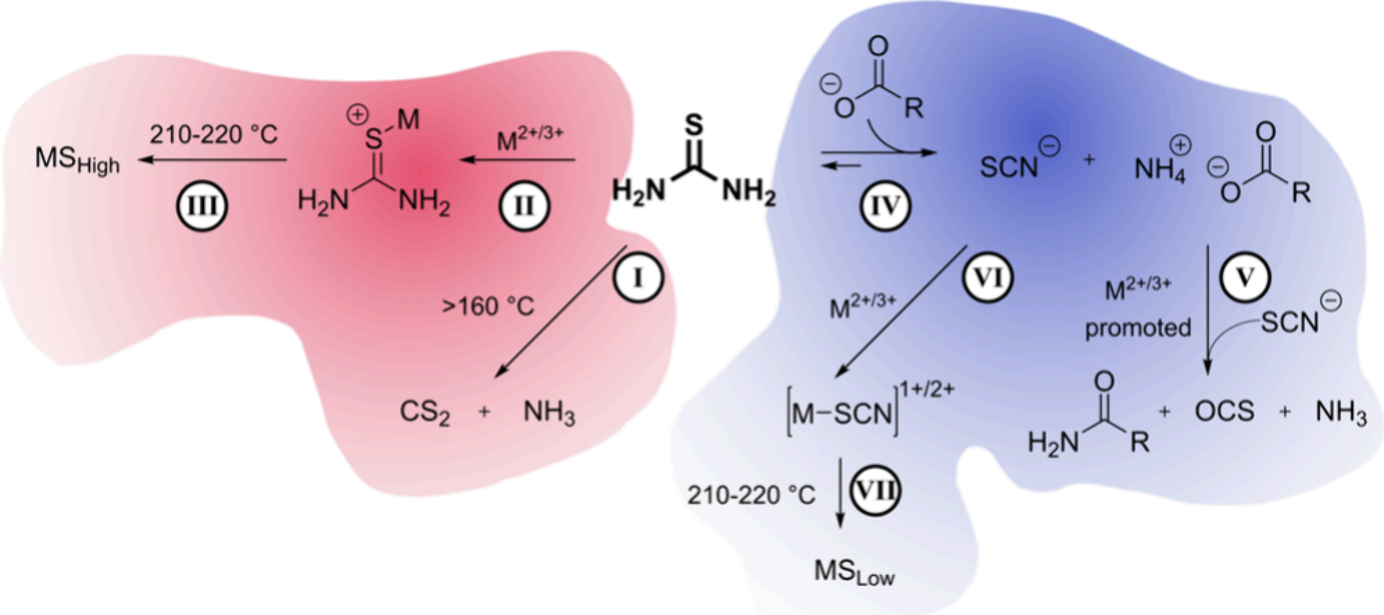
Oleic Acid Shifts the Solution Equilibria of Thiourea to the Much Less Reactive Thiocyanate Ion and Causes Sulfur Poor Metal Sulfides to Form Reprinted with permission under a Creative Commons 3.0 Deed License from ref (52). Copyright 2023 Royal Society of Chemistry.

Very recently, we studied the synthesis of iron, cobalt, nickel, and copper sulfides using thiourea and oleic acid as a coordinating ligand. It was hypothesized that oleic acid would bind the metal cations, slowing their reaction and lead to sulfur rich phases. Instead, the opposite occurred; high oleate concentrations led to sulfur poor phases for each metal. It was found that the oleate had side reactions with the thiourea changing the precursor to a much less reactive thiocyanate species (Scheme 3).^52^

Working toward full mechanistic understanding of phase control is pertinent to our complete understanding of nanochemistry. These studies rely on more traditional organic chemistry techniques such as ^1^H, ^13^C, and ^77^Se nuclear magnetic resonance (NMR), gas chromatography–mass spectroscopy (GC-MS), and even gas-phase infrared spectroscopy (IR) to track reaction progressions of the organic species. For the synthesis of metal selenides, ^77^Se NMR can be particularly helpful in determining intermedaites.^25,53,54^ The NMR solvent 1,2-dichlorobenzene-*d*_4_ is readily available and allows for reactions up to 178 °C. Therefore, *in situ* studies with heated probes of nanocrystal synthesis that happen at moderate temperatures are possible. Paramagnetic metals and the diversity and mixture of byproducts often make studies painstaking, but the challenges are not insurmountable.

## 4 Isolating Bond Dissociation Energy from Mechanism

As discussed above, a major challenge is that changing precursors to probe the effect of bond strengths often becomes convoluted with changing reaction mechanisms. A series of differently reacting, but similarly decomposing precursors are needed. Their reactivity should cover a large span of reaction rates to possibly access the full stoichiometric phase space. As an example where this approach has been employed, the Vela group studied the role of P(OR)_3_ reagents (R= Me, Et, *n-*Bu, CH_2_–*t*-Bu, *i*-Pr, Ph) in the phase-controlled synthesis of nickel phosphides and saw phase control between Ni_12_P_5_ and Ni_2_P based on the reactivity of the precursors.^55^ However, their study was not able to achieve any other of the many nickel phosphide phases, including Ni_3_P, Ni_2_P, Ni_5_P_4_, NiP, NiP_2_ or NiP_3_. Is the range of precursor reactivity too narrow to achieve all the phases, or are there other factors at play preventing access of these phases through changing the reactivity of the phosphorus source?

**Scheme 4:**
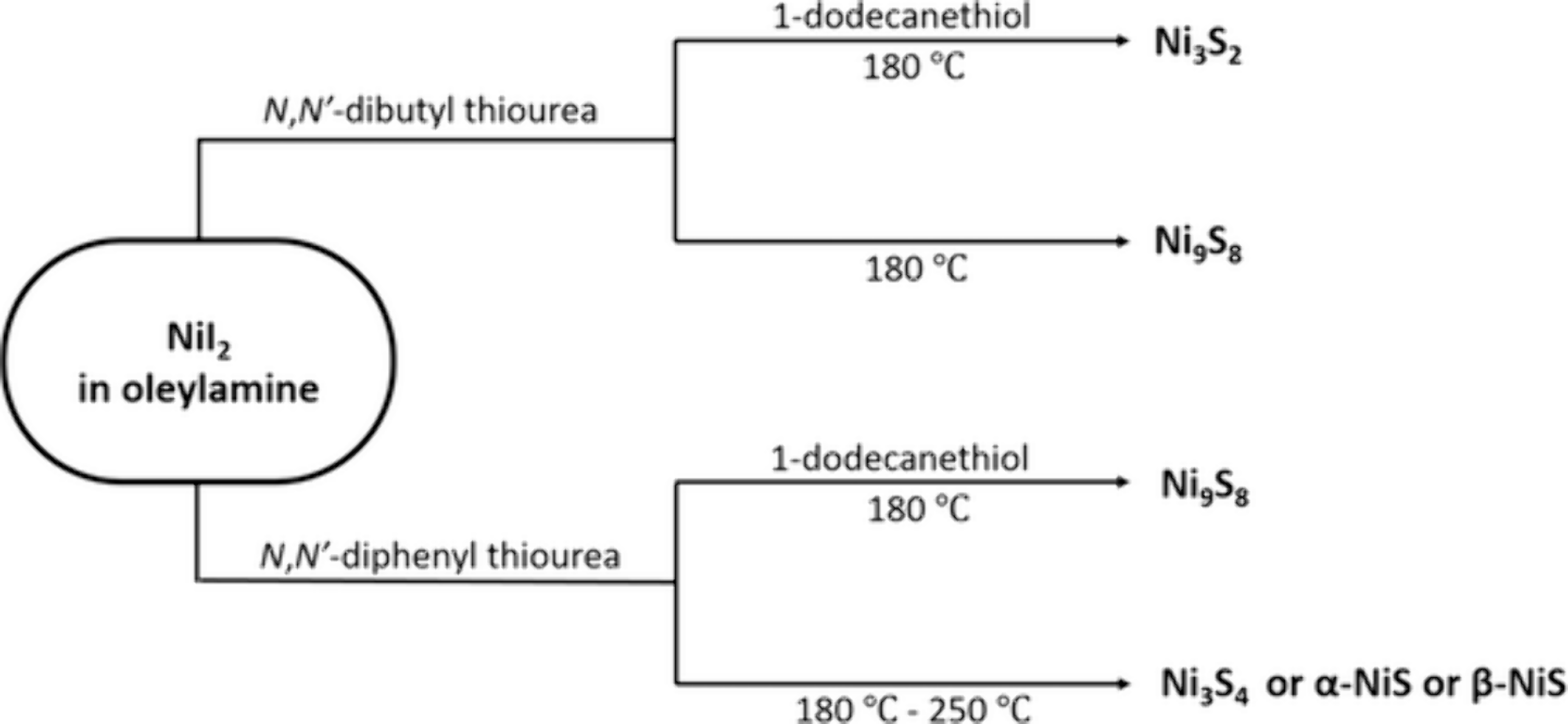
More Electron Rich Sulfur on a Substituted Thiourea of *N*,*N*′-Dibutylthiourea Gives Sulfur Rich Phases Compared to the More Electron Poor *N*,*N*′-Diphenylthiourea; Dodecane Thiol, While Nominally an Additional Sulfur Source, Seems to Hamper Sulfur Incorporation Reproduced with permission from ref (56). Copyright 2018 Royal Society of Chemsitry.

The Brutchey group performed a study on the phase control of nickel sulfides using substituted thioureas. *N*,*N*′-Diphenyl thiourea gave sulfur-rich phases (Ni_3_S_4_ and NiS), whereas less reactive *N*,*N*′-dibutyl thiourea gave sulfur-poor phases (Ni_9_S_8_ and Ni_3_S_2_). These results seem to agree with the idea that bond strength is an important determinant in phase control. Strangely, adding a second sulfur source, dodecanethiol seemed to have a secondary effect of slowing reactivity and giving sulfur-poor phases (Scheme 4).^56^ Even so, the phases of nickel sulfide observed do not cover the full breadth of the nickel sulfide phases space, and is notably missing NiS_2_.

What is needed are precursors that cover a very broad range of reactivity but have identical decomposition mechanisms to isolate kinetic considerations. The family of N-substituted thioureas developed by Mark Hendricks when he was part of Jonathan Owen’s group is ideal. Simply changing substituents on disubstituted thioureas altered the reaction rate in the synthesis of PbS by 4000-fold.^14^ By further changing the number of substituents (1, 2, 3, or 4), it can be postulated that at least several more orders of magnitude of rate control are possible. The Owen group has used this chemistry to precisely control the size of PbS,^14^ CdS,^13^ and ZnS^57^ nanocrystals (and PbSe^24^ with selenoureas) by altering the reaction rate of the thioureas with the metal carboxylate precursor. Since the thioureas should all have similar decomposition mechanisms, this library isolates only the kinetic factors in chalcogenide conversion rate that affect phase control.

**Figure 4:**
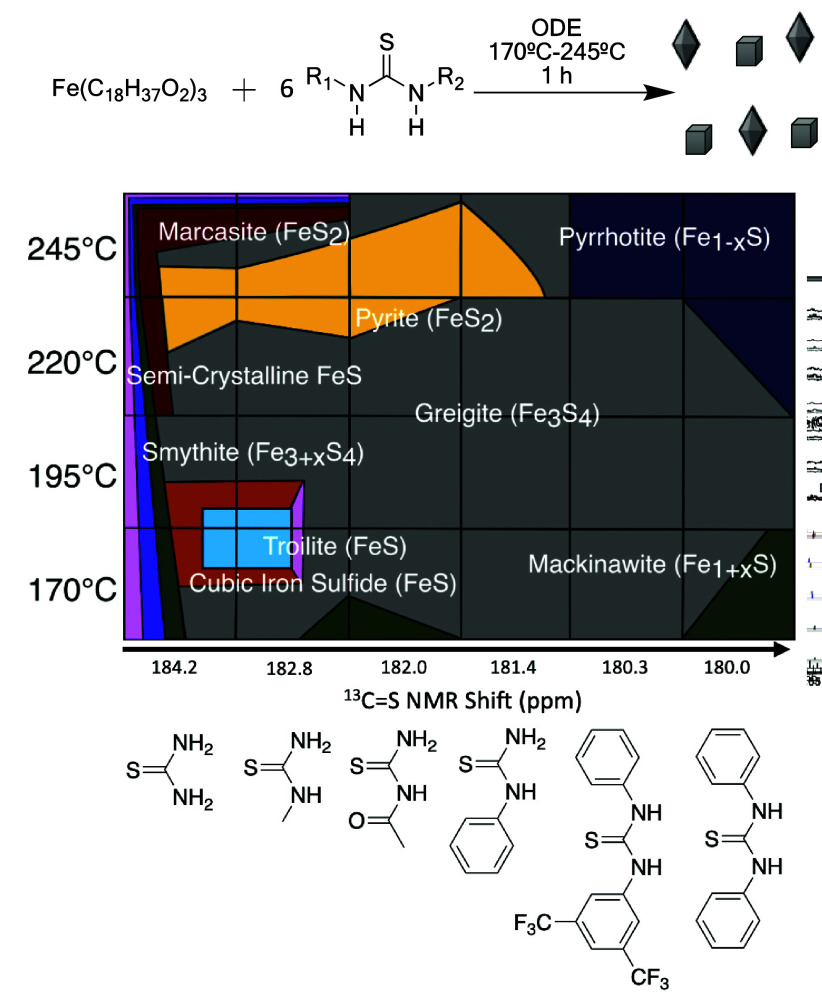
Above: Reaction of iron oleate with substituted thioureas to give iron sulfides. Below: A synthetic “phase diagram” showing the dependence on reaction temperature and thiourea reactivity on the crystalline phases formed. Areas in each block represent approximate ratio of the phases. All eight geologic phases were observed and one synthetic semicrystalline phase. Adapted from ref (58). Copyright 2023 American Chemical Society.

Using a truncated list of thioureas, our group systematically surveyed the chemistries of the iron sulfides (Figure 4).^58^ All eight geologic iron sulfides and a recently identified “unnatural” semicrystalline phase were identified. There are important implications to this observation: (1) Changing the inherent reactivity of a single precursor is sufficient to achieve all of the known phases. (2) Of all the phases that are seen, their interrelationships can be fully mapped out. (3) If this phenomenon translates to the other metals, then all the metal chalcogenides can be achieved, provided a sufficiently broad library of reagent reactivity is employed.

Careful consideration of the data allowed us to hypothesize that the path to thermodynamic stability of the iron sulfide phases is split into two paths, dictated by an approximate hcp or ccp lattice of the anions that is not easily crossed. Initial nucleation into an hcp phase or a ccp phase dictates which path is traveled.

**Figure 5:**
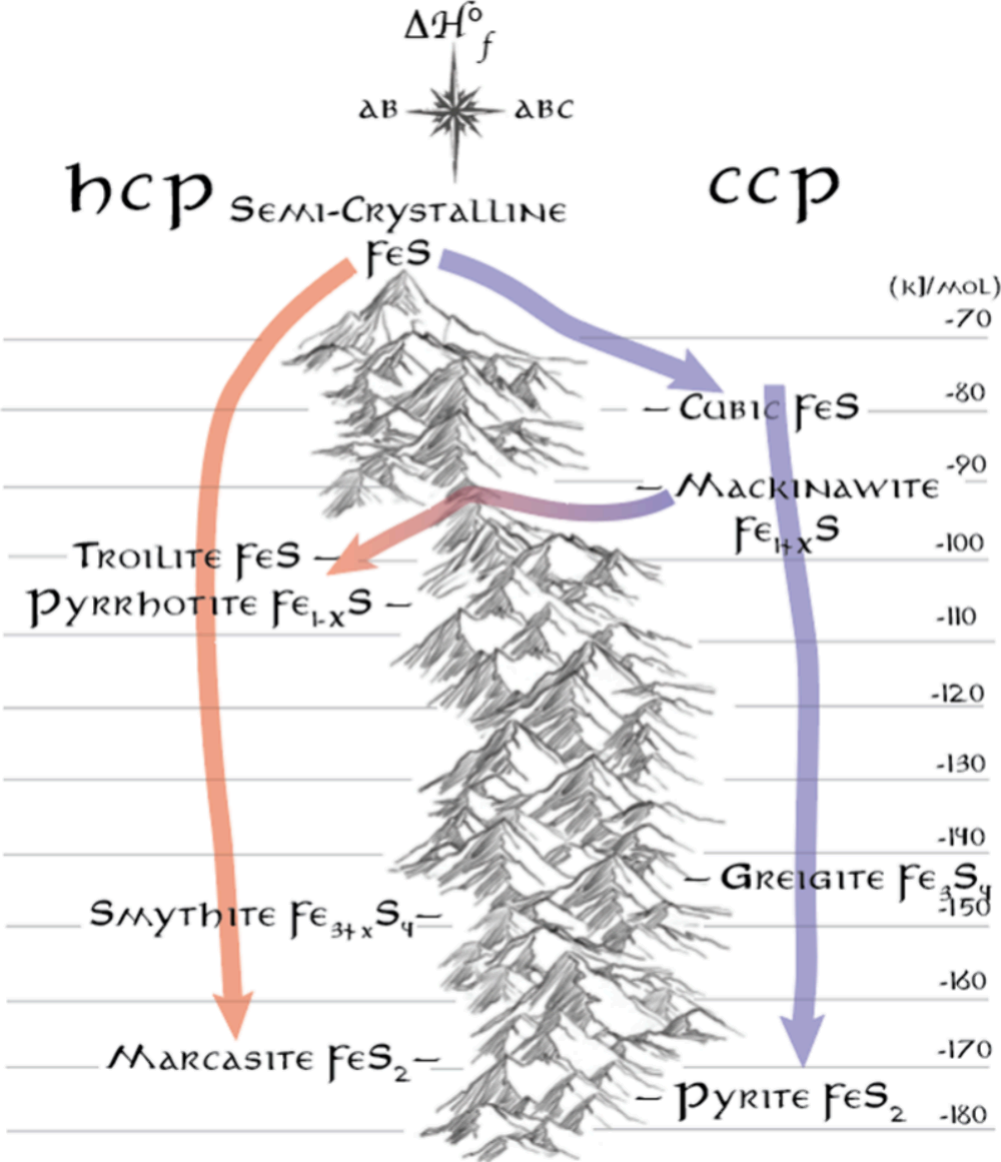
Colloidal synthesis of the iron sulfides follows predictable paths dictated by the approximate hcp and ccp stacking of the anions in the phases and the sulfur content. Only at one composition (∼FeS) and at sufficient temperatures (<220 °C) was a crossing between these anion stackings identified. Adapted from ref (58). Copyright 2023 American Chemical Society.

To visualize the phase progressions, we found it most instructive to draw it as an actual map with hcp and ccp “valleys” separated by a large activation energy “mountain range” (Figure 5).

The experiments suggested that very fast-reacting thioureas cause nucleation into both hcp and ccp lattices. Temperature and time dictated how far down the valleys the phase transformations occurred. Slow-reacting thioureas only formed ccp lattices and limited the formation of the most sulfur rich phases. At reactions over ∼220 °C, we realized there was a path between ccp FeS and hcp FeS. This is a highly unique way of thinking about phase transformations in synthesis, and it is a useful tool for simplifying and understanding relationships in ways not illustrated by traditional phase diagrams.

The biggest advancement was that we were able to use this map to rationally choose conditions that were selective for each of ccp Fe_1+x_S mackinawite, ccp Fe_3_S_4_ greigite, ccp FeS_2_ pyrite, hcp Fe_1–*x*_S pyrrhotite, hcp Fe_3+x_S_4_ smythite and hcp FeS_2_ marcasite. An example of the logic: metastable ccp Fe_1+x_S mackinawite was yielded by using superslow hexyl-phenyl-thiourea (off the chart of Figure 4 to the right) to avoid excess sulfur inclusion and transformation to ccp Fe_2_S_3_ greigite. Low synthetic temperatures were chosen to avoid transformation to the more stable hcp Fe_1–*x*_S pyrrhotite polymorph.

The mapping of the iron sulfides has achieved a main goal; we now have a new language and context for describing the terrain of phase control and make rational, rather than serendipitous, choices and discoveries. This new language needs to be tested by traveling though the other families of metal chalcogenides, especially those with more complex crystal structures.

**Figure 6:**
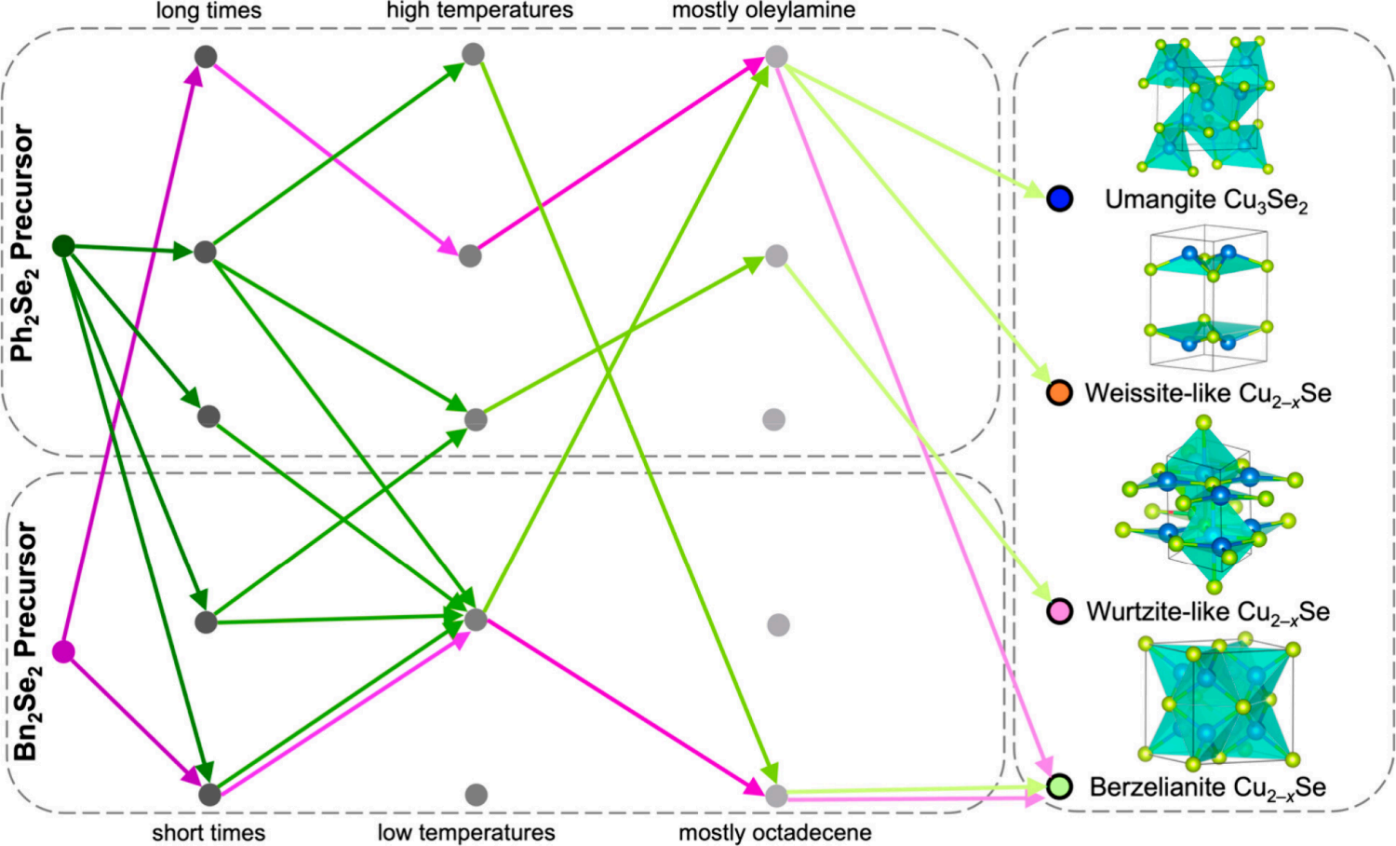
Decision tree for the synthesis of phase pure copper selenides predicted by a classification algorithm developed from a set of core experiments probing the phase space. Adapted from ref (59). Copyright 2023 American Chemical Society.

Concomitantly, the Brutchey group developed the idea of a reaction phase map, this time with a modified Design of Experiments approach^60,61^ to achieve phase selectivity in the copper selenides.^59^ In a series of 80 experiments, the team probed the role of temperature, time, and oleylamine: octadecene ratio, creating three-dimensional phase maps for two precursors of varying C–Se bond strength (Ph_2_Se_2_ and Bn_2_Se_2_) (Figure 6). Even though mixtures were often the product in the original 80 syntheses, the classification model allowed them to be able to predict the sometimes-tiny regions of the reaction space where phase pure products could be obtained. For example, 24.3 min, 223.5 °C and 4.7 volume% oleylamine in ODE were the predicted and experimentally confirmed conditions to give klockmannite (CuSe).

## 5 Phase Control of the Metal Pnictides

Compared to the metal chalcogenides, the phase control of metal pnictides in colloidal synthesis is far less studied even though they are important catalysts in oxygen reduction reactions and hydrogen evolution reactions.^62−65^ Pnictides have a comparable number of phases to the chalcogenides; for example, there are ten nickel sulfides,^66^ seven nickel selenides,^67^ and nine nickel tellurides,^68^ while there are four nickel nitrides,^69^ ten nickel phosphides,^70^ and five nickel arsenides.^71,72^ The lack of studies does not come from an absence of phases, but rather from synthetic challenges such as potentially dangerous reagents and extreme heat (700+ °C).^62,63^ Many metal pnictides are important catalyst materials,^73,74^ therefore, there is a large opportunity for growth in the area of phase control of the metal pnictides.

Metal nitride syntheses have been challenging because they require very high temperatures (700+ °C) in order to activate the nitrogen reactants.^62,63^ Commonly, inorganic amide precursors, such as amide salts, are used as nitrogen sources, but these ionic reagents lack solubility in the nonpolar solvents frequently used in high temperature colloidal syntheses. As an alternative, the Beaulac group found that alkylamides can be used in colloidal syntheses. They discovered that at 210 °C the oxidation of an alkylamide followed by the formation of a secondary imine generates the active nitride precursor, NH_2^−^_, for metal nitride formation.^63^ Because of colloidal complications, metal nitrides are more commonly synthesized through other noncolloidal methods such as ammonolysis,^75−78^ which is the formation of amines from gaseous ammonia or secondary amines,^78^ commonly done by taking powder metal precursors and treating them with ammonia gas at high temperatures in an autoclave or furnace.^76,77^ Additionally, a small number of metal nitrides has been synthesized using noncolloidal methods, such as solvothermal,^79^ heating in a furnace at high temperatures,^80^ thermal annealing, hydrothermal, and pyrolysis.^81^

The use of copper metal has allowed for lower synthetic temperatures (200–280 °C) specifically in colloidal syntheses of Cu_3_N, when using copper(II) nitrate with oleylamine or octadecylamine.^62,82−84^ The De Roo group explored the Cu_3_N system and elucidated the mechanism pathway from copper(II) nitrate and oleylamine and found that the nitrate is not the nitrogen source, but rather oxidizes the amine and aldimine, to which they draw comparison to InN like the syntheses mentioned above.^83^ The Moloto group also found that the decomposition time of the precursor affects the size and distribution of Cu_3_N nanocubes.^84^ Although we have yet to understand why the copper allows for lower temperatures, this is a good starting point for future studies. Similarly colloidal Ni_3_N has also been synthesized at lower temperatures (210–230 °C), using nickel acetate and oleylamine.^85^

Precursor chemistry of colloidal metal nitrides for group 4 to group 13 metals was explored in a recent review by the De Roo group, and potential mechanisms were discussed but have not been studied for most metal groups, outside of group 13.^86^ Based on their comparisons, they concluded that any convincing reports of a colloidal synthesis of group 4 and group 5 nitrides do not exist, as only nanopowders were obtained. Their forward-looking ideas to form 4 and 5 d colloidal metal nitrides includes starting with the metal in the correct oxidation state to skip a reduction step in the mechanism.^86^ Since the colloidal synthesis of nitrides is in its infancy, there has yet been no direct attention paid to phase control.

The colloidal synthesis of metal phosphides is more studied compared to the metal nitrides, although many phosphine reagents have potential to generate toxic gases as byproducts and are safety risks. Many synthesis methods for 3d metal phosphides include thermal decomposition of metal-phosphine complexes forming during the reaction of metal salts and trioctylphosphine.^87,88^ The Schaak group discerned the mechanism to form metal phosphides went through a metal nanoparticle intermediate before the phosphine was included into the product.^87^ This finding has allowed for controlled syntheses of 3–5 d metal phosphides. The M(0) nanoparticle intermediates also means that behaviors learned for chalcogenides will not work for pnictides using trioctylphosphine as a reagent.

There are only a few reagents used and methods known to moderate phosphorus reactivity in colloidal synthesis. The Brock group found that the quantity of trioctylphosphine or oleylamine, reaction time, heating temperature each had an influence on the phase of nickel phosphides.^89^ The Brutchey group explored methods to Ni_2_P nanoparticles and found a solvent dependence. In ODE with triphenyl phosphine as the phosphorus precursor, the resultant particles were a mix of Ni_12_P_5_ and Ni_2_P. When the solvent was replaced with the ionic liquid CYPHOS 104–which contains a n-tetradecyl phosphonium cation–the product was phase pure Ni_2_P.^90^ Hernández-Pagán, while in the Schaak group, found another way to form phase pure Ni_2_P by adapting work from Cossairt^91^ using tris(diethylamino)phosphine (TEAP) as the phosphorus precursor instead of the commonly used trioctylphosphine.^92^ Park et al. also explored changing the phosphorus source from trioctylphosphine to triphenylphosphite, TEAP, and tri-*n*-butylphosphine (TBP).^93^ Triphenylphosphite and trioctylphosphine gave mixed products, while TEAP and TBP gave pure FeP and Fe_2_P, respectively. The study also found that by increasing the duration of the experiment, the trioctylphosphine product would transition from Fe_2_P to FeP.^93^ Similarly, the group of Vasquez found that extended heating times and faster heating rates favored phosphorus rich FeP over a mixture of FeP and Fe_2_P when using TOP as a phosphorus source.^94^

The choice of metal precursor also influences phase formation *v*ia different reactivities of the metal or the attached anions. The Brock group found different reactivity in Fe, Ni, and Co precursors when forming M_2_P particles (at 300 °C).^95^ They used metal acetylacetonate salts and metal carbonyls with trioctylphosphine, oleylamine, and octadecene to explore monometallic and multimetallic transition metal phosphides. They found with the acetylacetonate metal precursors Ni_2_P would form but only Fe_3_O_4_ and CoO could be isolated, which was attributed to Ni(II) being more easily reduced than Fe(III) or Co(II). They also found that rate of phosphidation was much slower for Fe(CO)_5_ than for Co_2_(CO)_8_ and Ni(acac)_2_, overall showing metal precursor reactivity should be taken into account when synthesizing metal phosphides. When forming trimetallic phosphides, the order addition of the metals was key to success because of the different amorphous intermediates that formed.^95^ While the Brock group focused on reactivity of different metals, precursors of the same metal also show varying reactivity. The Sahu group explored phase control of tin phosphide by changing tin precursors, and was able to synthesize Sn_3_P_4_, SnP, and Sn_4_P_3_. Keeping the TEAP precursor constant, only the identity of the tin halides as well as different zinc halide precursors were chanced.^96^ The zinc halide was to aid the formation of Zn–N–P intermediates as activated precurors.^97^ They attribute their phase control to the relative bond dissociation energies of the halides, but the trend did not hold for SnI_2_ and ZnI_2_.

Other metal phosphide control studies include the Schimpf group, who showed they could synthesize Cu_3–*x*_P and demonstrated that the nucleation and growth reactivity can be tuned by varying the temperature and the oleylamine/P or P/Cu molar ratios.^98,96^ Other metal phosphides have also been synthesized colloidally, including CrP,^99^ MnP,^88^ Co_2_P,^88,91,100,101^ InP,^102^ Cd_3_P_2_,^91^ and Ni_2_P.^88^ As most of these syntheses do not explore phase control, and mainly focus on shape or growth there are more studies needing to be done.

Metal arsenides are limited in their studies, most of which are related to III–V quantum dots (QDs)^103^ or use noncolloidal syntheses such as ball milling.^104^ Some colloidal syntheses include work by the Jaramillo group who synthesized orthorhombic CoAs and MoAs crystals and hexagonal Cu_3_As crystals,^64^ and the Manna group who did the first colloidal NiAs synthesis.^105^ The Manna group also showed that control of the standard InAs synthesis could be added by mediating the reduction with ZnCl_2_, which improved the size distribution of the product, and allowed for a ZnSe shell.^106^ While these studies show a diversity of metal arsenides the studies do not include any phase control.

While many colloidal syntheses of all metal nitrides, phosphides, and arsenides have been reported, very little is understood about phase control in these systems. Concepts learned in metal phosphide studies should be applied to metal arsenide and nitride systems, to see how the concepts apply broadly as well as lead to a larger library of metal pnictides at our disposal. It is also unclear if the lessons learned in the chalcogenide family about bond energies and anion stacking will translate to the pnictides. A major challenge here is the lack of breadth in the reactivity of pnictide reagents that is not as broad as the chalcogens.

## 6 Polytype Control and Ghost Phases

The directed synthesis of nanocrystalline polymorphs (crystalline isomers) is particularly challenging because the synthetic handle of synthetic molar ratios is moot. Besides the known polytypic systems of the crystallographic record, the Materials Project has calculated a host of polymorphs across the binary metal chalcogenides and pnictides that have never been observed experimentally.^107^ Some of these “ghost phases” (as we call them in our group) even have calculated thermodynamic stabilities below that of the commonly observed polymorphs. This is a completely untapped resource of unique materials and material properties. Even so, the Materials Project is still incomplete in identifying ghost phases in all element combinations. Our group alone has developed direct syntheses for two novel phases including wurtzite Cu_2_Se^108^ and pseudocubic Cu_1.5_Te.^109^ The Schaak group has also synthesized a novel weissite-like phase of Cu_2–*x*_Se.^65^ The possibilities are there, if only we could find the conditions to prepare the ghost phases.

The most well studied polymorphic system in colloidal synthesis is CdSe, which exists in either a thermodynamic hexagonal wurtzite (WZ) or a metastable cubic zinc-blende (ZB) phase. There are two common approaches employed to obtain polytype control, but as you will read, these are not mutually exclusive.

### 6.1 Kinetic Argument: Go Slow to get Metastable Phases

In 1897, Wilhelm Ostwald observed that metastable phases tend to form first after nucleation, before conversion to the thermodynamic phase.^110^ While colloquially referred to as the “Rule of Stages,” this was based on a set of observations, and he did not codify it as a principle. Since the phases presumably form in sequence, this idea has long been employed to capture metastable phases using reaction kinetics; however, just because a phase is metastable does not mean it has a lower energy barrier of formation. Thus, the question remains: why do these phases form first?

At the small sizes shortly after nucleation, the surface area-to-volume ratio is massive, so surface chemistry plays a large role in determining phase. As such, the fastest way to achieve stability at the onset of nucleation is for the monomers to arrange themselves in a way that has minimal surface energy.^111^ Ceder and colleagues of the Materials Project postulate that metastable phases are observed first because they are actually the thermodynamic phase at small sizes due to minimized surface energy, and the existence of these phases in larger sizes occurs through retention and growth of that crystal lattice with what they call “remnant metastability”.^112^ In support of their principle, the Ceder group has shown that marcasite FeS_2_ (which is metastable in the bulk) is actually lower in energy than its pyritic counterpart at acidic pH due to minimization of surface energy at small particle sizes. The lowered surface energy leads to exponentially faster rates of nucleation for marcasite over pyrite.^113^ In the hunt for conditions to yield ghost phases, the researchers propose that the most important factor will be finding conditions that make small nuclei of the ghost phases the thermodynamic preferred phases to allow for preferential nucleation.^114^

**Figure 7:**
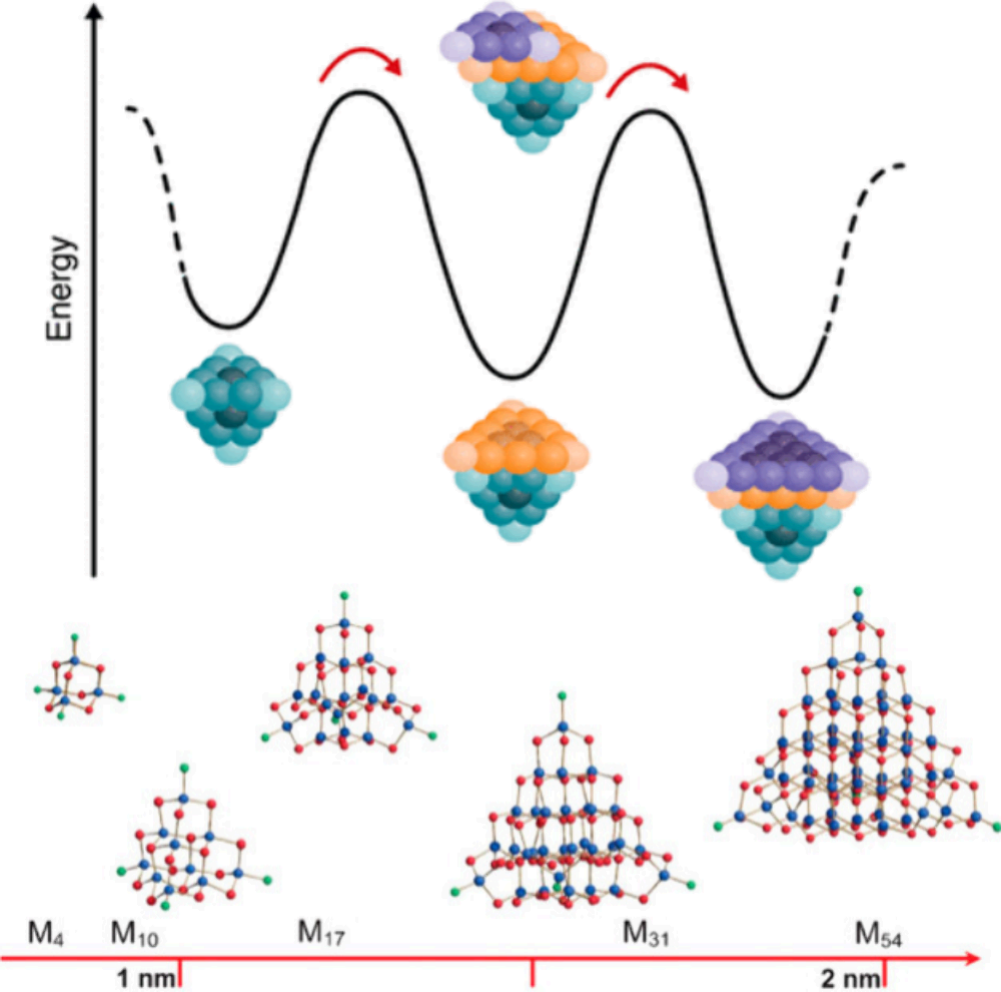
Top: Schematic of the thermodynamic landscape of magic sized clusters of CdSe showing the addition of close packed layers of Se^2–^,^114^ which are presumed to be the intermediates en route to zinc blende CdSe nanocrystals. Adapted from ref (114). Copyright 2021 American Chemical Society. Bottom: Known clusters of CdSe and CdS that are pure adamantyl (M4 and M10) and others with adamantantyl cores and barrelane shells or vertices (M_17_, M_54_, M_54_). Blue: Cd, red: S or Se, green: ligand. Adapted with permission from ref (115). Copyright 2009 Wiley.

As direct examples of Ceder’s postulate, magic-sized clusters (MSCs) are being increasingly recognized as intermediates in colloidal synthesis. These small molecular structures (less than 2 nm) are denoted as “magic” due to their exact atomic compositions,^116^ and they have been observed as quantized intermediates in the formation of nanocrystalline Au,^117^ InP,^116^ CdSe,^118^ PbS,^24^ ZnS,^119^ CdS,^119^ CdTe,^119^ ZnTe,^119^ among others. MSCs represent local thermodynamic minima in the formation of solids (Figure 7).^114^ Because these MSCs are the building blocks for bigger NCs, the structure of the MSC can serve as a template for the resultant phase. Corrigan and colleagues pointed out that experimental structures of MSCs can be made of pure adamantane-like structures which are a microcosm of the cubic zinc blende phase. Other MSCs have adamantyl cores but with shells that have barrelane-like cage moieties which mimics the hexagonal wurtzite phase.^115,120^ As a result of this templating effect, MSCs have been exploited as single source precursors for the precisely controlled growth of nanocrystalline CdSe, ZnSe,^121^ InP,^122−124^ and InAs;^125^ such a method permits high levels of size control, narrow size distributions, phase purity, and large-scale production of NCs. Additionally, this method gives a route to high-quality QDs at low reaction temperatures (typically <200 °C) because MSCs seed the growth of QDs, thus eliminating the need for a nucleation step.^126^

While some metastable phases can form first because of their remnant metastability, upon growing to larger sizes where surface energy is less important, it will become more energetically favorable to rearrange to the bulk thermodynamic structure. In 2012, the Strouse and Tilley groups hypothesized that nucleation of NCs under fast reaction kinetics causes the formation of vacancies and faults in the crystal structure of the nucleated metastable phase; these defects amass and ultimately catalyze collapse into the thermodynamically preferred phases during growth. By intentionally changing reagent concentrations to slow down reaction kinetics, they exploited Ostwald’s Rule of Stages to achieve 14 nm metastable ZB CdSe NCs, much larger than any previously reported synthesis at the time. They attributed the success of these syntheses to the slower reaction kinetics which led to the formation of more perfect NC structures that lacked the defects necessary for thermodynamic phase collapse.^127^

**Figure 8:**
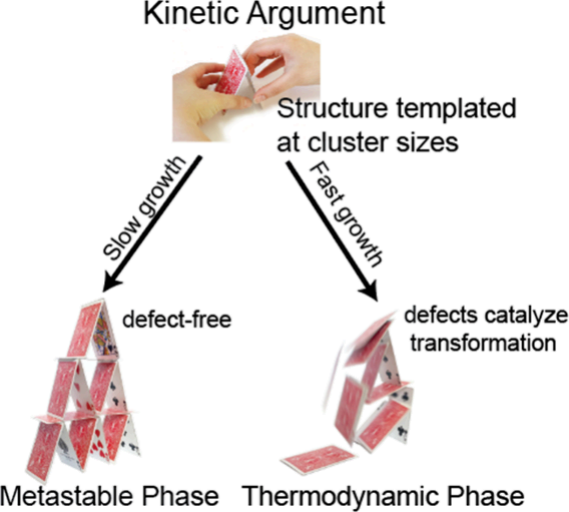
Kinetic approach to controlling phase in crystal growth. The argument suggests that metastable phases form first, but their collapse to their thermodynamic phases is catalyzed by defects. Slow, careful growth to prevent defects traps the metastable structure as it grows to larger sizes.

In short: the hypothesis is that slow reaction kinetics lead to defect-free crystals that retain the structures of nucleated metastable phases (Figure 8). Thus, we call intentionally using slow reaction kinetics to trap a metastable crystal structure the “kinetic argument.″ It should be noted that going slow to get the metastable product is in contrast to the approach employed by molecular chemists, who quench reactions early to isolate kinetic products.

In another example, metastable wurtzite-like Cu_2–*x*_Se was prepared using didodecyldiselenide as a precursor; dodecylselenol instead gave the common thermodynamic cubic product. The selenol reagent creates an intermediate Cu-selenoate complex, which greatly lowers the C–Se BDE and makes it more reactive. The didodecyldiselenide does not make such an intermediate, and so reacts much more slowly to give a copper selenide. In this case, the slow reaction kinetics afforded by the diselenide allow for growth and retention of the metastable Cu_2–*x*_Se phase.^108^

It appears that dichalcogenide precursors tend to give metastable products. This trend has been seen for sulfides,^42^ selenides,^42,43,108^ and tellurides.^26,109^ The chemistry of these precursors lends them to reactivity at moderate temperatures, hindering thermal activation of transformations from metastable to thermodynamic phases. Second, as hypothesized in the case of Cu_2_Se,^108^ their decomposition may give slow, careful deposition of monomers on growing nuclei through their particular reaction mechanisms.

Again, one must be careful in assuming kinetics are isolated from mechanism in a given study. The Vasquez group examined aqueous chemical bath deposition of CdS and found that fast thiourea decomposition correlated with the formation of metastable zinc blende phase. High pH caused fast thiourea decomposition but also forced the reaction to undergo a “cluster hydroxide” mechanism with Cd(OH)_2_ in solution. Lower pHs caused very slow thiourea decomposition, but also changed the mechanism to “ion-by-ion” since the active cadmium species is Cd^2+^. At the lower pH the thermodynamic wurtzite phase dominated.^128^

The “kinetic argument” appears not to be universal. In possible contradiction, Hendricks and Owen used a series of substituted thiocarbonates, dithiocarbonates and thioureas to influence the kinetics of a CdS synthesis, (which has similar polytypes to CdSe) by 5 orders of magnitude. In all cases, the metastable zinc blende structure resulted, and polytypic control was not achieved.^14^ Furthermore, the Owen group has provided increasing evidence that monomer diffusion kinetics, rather than precursor availability, are integral in NC nucleation and growth; Abécassis et al. employed a series of the same thioureas in the synthesis of PbS NCs, yet observed a singular growth rate constant for all thioureas despite drastic differences in *in situ* monomer availability.^129^ Nucleation and growth of PbS NCs were best modeled by surface-reaction limited growth, with monomer penetration of the oleate ligand shell as the rate-determining step.^129^ Nonetheless, further studies into how ligand diffusion kinetics impact nanocrystalline phase are necessary.

### 6.2 Surface Thermodynamics Argument: Ligand Head Groups

**Figure 9:**
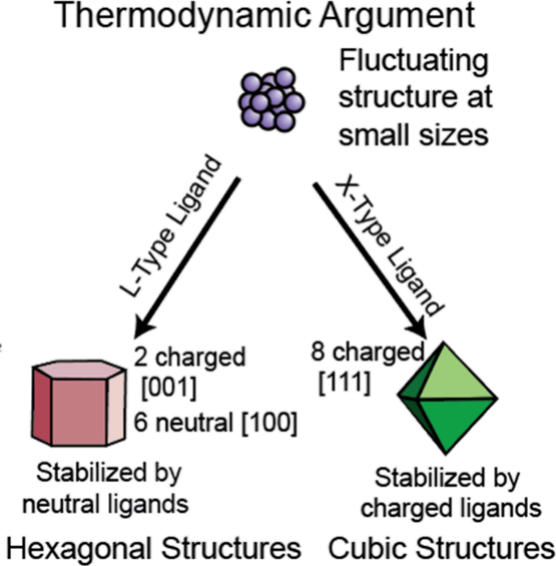
Thermodynamic approach to polytype control in colloidal nanocrystal synthesis. The thermodynamic argument suggests that high surface area:volume ratios and appropriate ligation make metastable phases thermodynamically preferred products at small sizes.

Given the very few unit cells in a small nanocrystal or “nucleus,” Rosenthal et al. suggested that the enthalpy difference between the ZB and WZ phases of CdSe can be smaller than the thermal energy; as a result, ultrasmall, 1.5 nm CdSe particles have an indiscriminate and fluctuating structure.^130^ Gao and Peng later made the argument that for 2 nm CdSe the difference in enthalpy between the ZB and WZ structures (∼0.11 eV) is negligible compared to the surface interactions. A single hydrogen bond, for comparison, is 0.21 eV, so surface ligand binding effects dominate.^111^ This argument is consistent with the previous observations that anionic X-type ligands (e.g., oleates, phosphonates) stabilize the ZB phase, whereas neutral L-type ligands (e.g., phosphines, amines) lead to the WZ phase.^131,132^ Huang, Talapin, and Kovalenko hypothesized that the anionic ligands make strong bonds to the eight cationic [111] facets of the cubic ZB structure, while the hexagonal WZ structure can only present two such charged [001] facets. On the other hand, neutral L-type ligands favor the WZ phase with its predominance of neutrally charged surfaces^133^ (Figure 9).

More recently, Zamkov and colleagues found that the aggregative growth, or growth of large crystallites via coalescence of smaller particles, in CdX (X = S, Se) NC systems was driven by thermodynamic factors; under L-type ligation, cubic ZB structures converted to hexagonal WZ structures while ZB structures were retained under X-type ligation.^134^ Similarly, Huang and colleagues utilized *in situ* wide-angle X-ray scattering (WAXS) to observe the phase transition of CdSe QDs annealed in the presence of different coordinating solvents and found that annealing WZ CdSe with propylphosphonic acid causes a phase change to the cubic ZB phase, while the reverse reaction was not impacted by the presence of propylphosphonic acid.^133^

Under the view of Ceder’s remnant metastability,^112^ these surface ligations provide the surface conditions to stabilize the metastable phase at small sizes. What is particularly interesting is that the surface stability seems to extend well past “nucleation” sizes, as the Zamkov group has prepared 50 nm particles using a ligand mediated aggregation approach.^134^ It appears, therefore, in ligand-driven ripening processes and in nucleation, one must consider only the local thermodynamic energy landscape around monomers that are being deposited on growing surfaces, as this outweighs global thermodynamic pressures of the whole crystal. But this is an idea that needs to be thoroughly tested.

Unfortunately, the surface thermodynamics argument has not been well tested or developed beyond CdSe, and this is a major blind spot that should be addressed. Other polymorphic systems should be subject to the same experimental approaches to see if the kinetic and/or thermodynamic arguments hold true. Copper selenide, in particular, provides an interesting data point for such studies, as in direct contrast to CdSe, the wurtzite-like phase is the metastable polymorph of Cu_2_Se, and the cubic phase is thermodynamic.

## 7 New Approaches

### 7.1 Phase Templating

**Figure 10:**
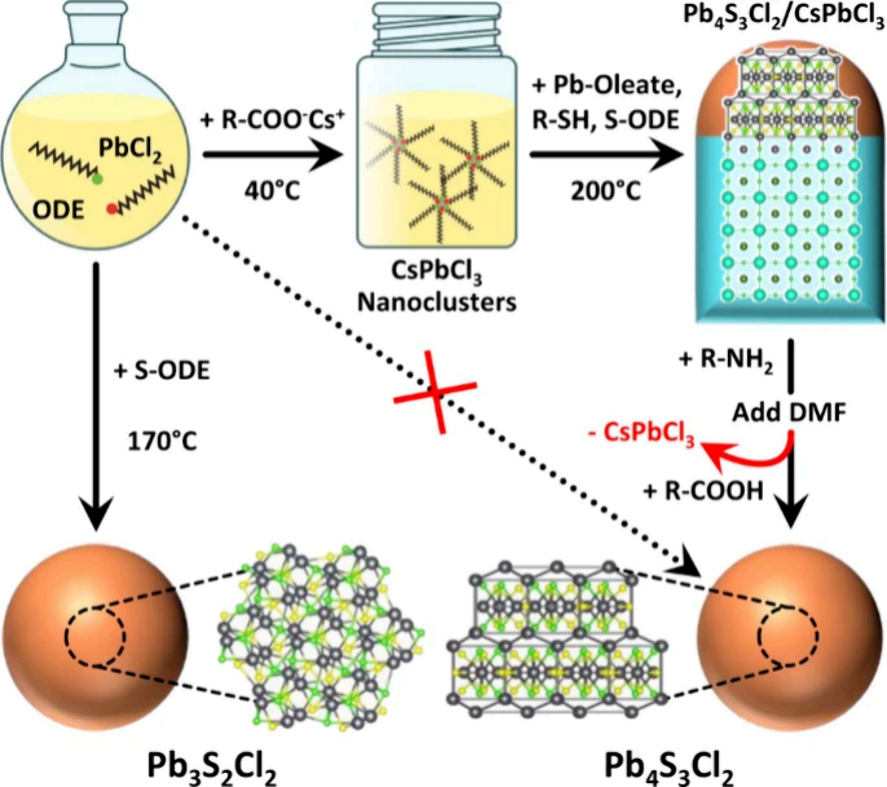
Example of phase templating to obtain the metastable Pb_4_S_3_Cl_2_. Reprinted with permission under a Creative Commons CC BY License from ref (135). Copyright 2022 Nature Publishing Group.

An exciting new idea in phase control is the use of sacrificial crystals for heteroepitaxial growth. The Manna group found that synthetic conditions to yield Pb_3_S_2_Cl_2_ would instead yield Pb_3_S_3_Cl_2_ if CsPbCl_3_ was present, a phase that was not known to the literature. The sulfonamide perovskite heteroepitaxially grew off the template and maintained the structure of the cationic lattice. In this case, the template CsPbCl_3_ was easily removed because it is water-soluble while the chalcohalide was not (Figure 10).^135^ Other explorations of heteroepitaxial growth of chalcohalides are ongoing by the Pradhan group.^136^

Phase templating is an opportunity for machine learning to comb through the crystallographic database to find combinations of materials that may grow heteroepitaxially on one another. Any visit to a geology display demonstrates heteroepitaxial growth on the macro scale, and so there are opportunities to find examples inspired by nature. It is such a nascent approach that there is be much to be discovered about the extent of the possibilities, the roles of ligands and ionic vs polar covalent materials.

### 7.2 Stabilization with Impurity Metals

If one starts closely examining the mineralogical literature, common themes in geology emerge of impurities of a second or third metal being highly associated with certain mineralogical phases. This, of course, is a major approach of metal alloying: altering the metal ratios to obtain different metal packings with improved strength (body centered cubic phases) or ductility (close packed phases). Ion stabilized phase is also employed in biomineralization processes. Trace nickel ions influence the phase of precipitated calcium oxalate, which has important implications for kidney stones.^137^ In compound polar covalent and ionic materials, how impurity metals stabilize metastable structures is not well understood in many cases, and very rarely employed as a tactic in nanocrystal synthesis.

There are exceptions, including the Plass group, who were able to stabilize of a rare tetragonal copper sulfide using iron.^138^ The use of metal impurities for the stabilization of chosen phases could be further employed by taking a geomimetic approach and searching the mineralogical databases for common combinations.

## 8 Hunting Ghosts

As materials chemists, we have well established tools for characterizing nanocrystalline materials including powder X-ray diffraction (pXRD), electron microscopy, and steady state absorbance and fluorescence spectroscopy for plasmonic and semiconductor materials. However, there are challenges that inhibit facile characterization of phase control phenomena: the solving of novel crystal structures of small crystals, and experiments that provide second or subsecond temporal resolution of transient crystallite characterization *in situ*.

### 8.1 Solving Novel Structures

pXRD is the most prominent characterization technique for identifying and quantifying crystalline materials. Not only is its ability to characterize crystalline phases unmatched, but it also offers additional information about crystallite size, preferred orientation, and lattice strain.^139^ Unlike transmission electron microscopy (TEM) which only measures a few particles, it characterizes milligrams of material at a time, and is an excellent technique for identifying impurities. Finally, the ease of access and use of pXRD characterizations makes it easily accessible to most nanocrystal chemists. pXRD is the go-to first technique for characterization when phase is important. Most pXRD characterizations are postsynthetic, but there are few studies showing that this technique can be used *in situ* using intense synchrotron X-ray sources.^140^

Despite these advantages, pXRD is an imperfect technique. In samples with multiple phases present, the limit of detection for mixed crystals is about 4 wt % of the sample.^141−143^ It should also be noted, that peak width is dominated by the largest crystals, making Scherrer analysis to obtain crystallite size difficult for polydisperse samples. Furthermore, Sherrer analysis is only qualitative above about 60 nm.^142^ It is exceedingly difficult to solve powder patterns and near impossible for nanocrystalline materials with broad peaks. The exceptions are when a structural analogue is identified by sheer experience looking at patterns, with examples like wurtzite-like CuInS_2_,^144^ wurtzite- like CuInSe_2_,^145^ wurtzite-like Cu_2–*x*_Se,^146^ and weissite-like Cu_2_Se.^65^ Our group has left several crystal structures unsolved, and projects (hopefully temporarily) abandoned due to the limitations of diffraction techniques. The ability to identify and solve novel crystal structures of nanosized crystals with ease is a monumental challenge. The outlook of this field will be greatly impacted by the new up and coming techniques that are highlighted here.

While pXRD is a bulk technique, selected area electron diffraction (SAED) phenomena in the transmission electron microscope can be used to identify crystals in mixtures. It can be particularly helpful when after pXRD one is unsure if there are two phases, or one complex phase.^147^ When high resolution images yield lattice fringing on individual crystals, Fourier transforms of lattice fringing work as a *de facto* diffraction technique. Alternatively, true selected area electron diffraction can be performed on individual crystals or on groups of crystals. The Golan group used these techniques to obtain a full structure discernment of novel π-SnS phase, discerning it from a mixture of α-SnS nanocrystal and π-SnS phase.^148^ While electron diffraction is mostly commonly performed by hand concomitantly with imaging, electron diffraction is becoming automated.^149^

**Figure 11:**
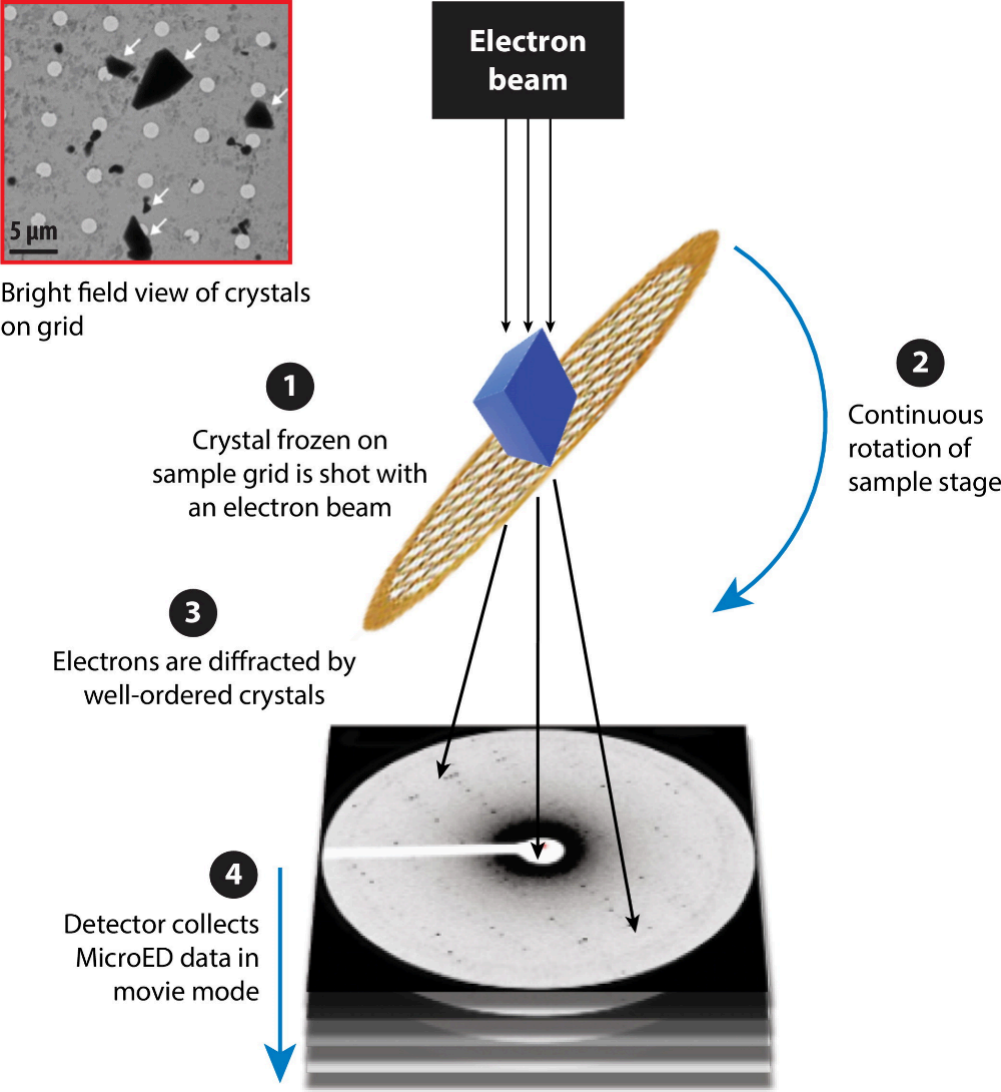
Concept of three-dimensional electron diffraction (3DED) to obtain single-crystal patterns of micron and submicron crystals. Adapted with permission from (154). Copyright 2021 Annual Reviews.

Three-dimensional electron diffraction (3DED) is a series of closely related techniques that can acquire a single crystal spot pattern that is analogous to that of traditional single crystal XRD.^150^ Single crystal X-ray diffractometers have the source and detector circle around a single crystal, while in MicroED (as an example of a 3DED technique), the TEM grid is rotated while the beam stays steady and data is collected on the spot pattern that results from a single crystal. The collected patterns can be solved similarly to a single crystal pattern (Figure 11). There are a few pioneering cases of 3DED being used to solve novel crystal structures on the nanoscale,^151^ but so far it is a vastly underutilized technique in materials science.

Electron diffraction is more sensitive to low mass atoms than X-ray diffraction, and it is possible to gain meaningful data from light elements such as carbon. For this reason, 3DED is being deployed for solving crystals structure of small molecules and proteins without needing to prepare a macro-sized crystal.^152^ The technique therefore can sometimes be found associated with cryo-EM rather than materials characterization facilities. Rigaku and JEOL are teaming up to develop a single crystal dedicate MicroED instrument.^153^

The challenge with 3DED is that the technique requires as an ultrastable goniometer, or else the crystal of interest will drift out of view during data collection. In practice, the technique seems to require crystals of 40–100 nm or more. It is still quite large for many inorganic colloidal systems of novel phases. The technique is still not user-friendly, requiring expertise in sample acquisition and a patient molecular chemistry colleague willing to teach us how to solve single crystal patterns.

A possible complementary technique is atomic electron tomography, which can give the 3D coordinates for individual atoms at near-atomic resolution. Billinge et al. debuted this technique by creating a 3D rendering of a gold nanoparticle,^155^ and later the Zhou group, showed that one can even view the different facet growths of Pt nanoparticles during nucleation.^156^ Since very small crystals are needed and desired, there is a great potential for this technique to provide some hints as to atom placement in real space before trying to solve powder patterns. Again, this is a very specialized technique requiring some of the most powerful scanning transmission electron microscopy (STEM) instruments currently available.

Nonetheless, this new family of ED techniques will quickly improve with time, and nanocrystal chemists should jump on this opportunity.

### 8.2 Time Resolution

The Ostwald rule of stages suggest that some of the ghost phases may be transient in the growth of nanocrystals. For this reason, *in situ*, time-resolved diffraction techniques would be very useful.

Heated chambers are relatively common in pXRD, but the ability to perform experiments in solution are difficult because the concertation of particles is too low. Solution phase TEM experiments have been performed watching nucleation and growth,^141,157−159^ but the high electron dose means that the chemistry is very reducing and the types of reactions that can be suited are limited and may not reflect in-flask phenomena. As of this time, for most researchers in this field, “at home” *in situ* experiments following crystalline phase remains a dream.

Synchrotron light sources provide a strong enough signal to allow for *in situ* measurements of crystalline phase in colloidal synthesis, typically using small-angle and wide-angle X-ray scattering (SAXS and WAXS). WAXS probes length scales indicative of atomic crystalline order, whereas SAXS can help measure particle size, size distribution and any ordering on the particle scale. Banfield et al. used *in situ* temperature variable SAXS and WAXS measurements to understand how water can drive structural phase transformations of ZnS nanocrystals.^160^ Owen et al. have recently used synchrotron techniques to study the nucleation behavior of PbS.^161^ de Mello Donega et al. used SAXS to study the stacking of lamellar copper–dodecane thiol sheets to Cu_2–*x*_S nanosheets and spheroids.^162^ SAXS/WAXS synchrotron experiments offer good time resolution and ability to monitor phase transformations *in situ*, but it appears it works best for very carefully designed systems and reactions that react over minutes rather than seconds. The requirement of a synchrotron source means *in situ* WAXS and SAXS remains mostly inaccessible to many nanocrystal chemists for routine study.

Often forgotten are techniques used by our colleagues in other fields of chemistry for *in situ* measurements from which the phase or mechanism can be indirectly inferred. Steady-state absorption spectroscopy has been used to distinguish between 2 nm wurtzite and zinc-blende CdSe in liquids.^163^ The Cossairt group used distinctly molecular-looking absorption patterns to identify cluster intermediates in nanocrystal growth.^124,164^ Absorption spectroscopy is a technique available to almost every chemist at their home institutions, and so it could be applied more broadly. Similarly, nuclear magnetic resonance (NMR) is underutilized to determine the organic mechanisms that preclude nanocrystal formation. It is not terribly uncommon to find NMRs that are designed to heat samples and so it is possible to do *in situ* studies of nanocrystal syntheses. Unfortunately, NMR is a relatively long technique, with sampling time on the order of seconds to minutes which makes it difficult to study burst events. While the instrumentation exists and has been applied to other fields of chemistry, we are unaware of *in situ* Raman or Infrared spectroscopy used to study nanocrystal syntheses *in situ*. These spectroscopies can be used to distinguish phases. For example, Hofmann et al. used Raman spectroscopy mapping to show different domains and phase junctions of pyrite and marcasite materials.^165^

In summary, while chemists have creatively used synchrotron SAXS and WAXS, as well as more common spectroscopies to follow nanocrystal growth *in situ*, the temporal resolution is insufficient for many nanocrystal syntheses. Only one *in situ* technique, WAXS, can directly measure prove crystal structure, and it is limited to selective synchrotron sites.

## 9 Conclusions

A visit to a geology exhibit at a museum is always awe inspiring for a chemist. To make the glorious fist sized crystals on display, geology has assembled with precision and perfection individual atoms of a number so large that that it has 23, 24, or even 25 digits. It is mind boggling to think of it.

Equally wonderful is to imagine the event that gave rise to that crystal; somewhere in the core or at the base there are the first tens of atoms that found each other and assembled into a nanoscopic crystallite, perfectly aping and expressing the symmetry of the macro-sized crystal that was to come.

Questions arise that are fundamental to what we do as chemists. Why this particular arrangement of atoms in this particular ratio? Why this arrangement of atoms in this environment and a different arrangement found elsewhere made of the same elements? How do the properties of those crystals depend on their atomic identities and arrangement in space?

Armed with Schlenk lines, glove boxes, diffractometers, and electron microscopes, synthetic nanocrystal chemists have in essence, with the aid of surface binding ligands, been capturing moments right after nucleation; yet we are deeply dissatisfied. Because we cannot yet observe directly the “Big Burst” event, if there even is one, we do not yet know how or why certain crystal phases form under certain conditions. Surprisingly, this is a problem the geologists have not satisfactorily answered either.

In the past few years, some important trends are becoming clear. (1) Phase space in the metal chalcogenides and pnictides is highly complex, yet organochalcogenides and organopnictides reagents are a powerful way to moderate reactivity and influence phase. (2) Any experimentation and discussion about phase control must carefully separate the factors of how fast a precursor decomposes from the reaction mechanism by which it does so, especially on metal centers. (3) Despite the complexity, there are logical paths through the phase space by which crystallites transform from one phase to another. The paths are related to crystallographic similarities between phases. (4) Reactions with slow reaction kinetics at moderate temperatures facilitate the isolation of metastable polymorphs. (5) The geologic and crystallographic record is incomplete—even for binaries. Colloidal syntheses have and will continue to achieve new “unnatural” metastable phases that will provide new material properties.

Despite these advances, phase control is still very much a black box in nanocrystal syntheses. (1) More careful examination of the molecular transformations that preclude nanocrystal formation is needed. (2) While understanding phase control in the metal chalcogenides is quickly moving forward, the pnictides are sadly neglected, in part because the available organopnictide precursor scope is limited. (3) We need to test long-held theories about polytype control in CdSe with other materials. (4) Better techniques and methods for solving novel crystal phases at extremely small sizes are greatly needed to discover new materials. (5) We need improved and more easily accessible techniques that can follow nanocrystal nucleation and growth *in situ* to understand fundamental phenomena of phase control.

Unfortunately, many of the factors that are being found to influence phase are also key to controlling size and morphology, including reaction rates and the presence of coordinating ligands. It is all intertwined. Controlling all three concurrently presents a large challenge. At this point, we need studies that control each independently. Factors which control size and shape have been well developed over the last two decades; phase control is the one factor that remains understudied. It is only after we understand phase control alone, that we can attempt to control size, shape and phase at the same time.

Phase control is an exciting area for work and discovery in part because there is the opportunity to be the first to synthesize crystals never seen before, and potentially be the discoverer of a material that will solve a pressing technological challenge. Yet there is a far more important reason to master crystallization and phase control phenomena. Uncovering the fundamental principles of crystallization and phase control will provide an evergreen benefit to all that make or employ crystalline materials across the periodic table. Long after the current ephemeral blooms of individual nanomaterials fade, to be supplanted by new crystalline materials and challenges in nanoscience, the fundamental principles of phase control will live long after.
